# Premature Infants Have Normal Maturation of the T Cell Receptor Repertoire at Term

**DOI:** 10.3389/fimmu.2022.854414

**Published:** 2022-05-30

**Authors:** Sarah U. Morton, Maureen Schnur, Rylee Kerper, Vanessa Young, Amy E. O’Connell

**Affiliations:** ^1^Division of Newborn Medicine, Boston Children’s Hospital, Boston, MA, United States; ^2^Department of Pediatrics, Harvard Medical School, Boston, MA, United States; ^3^Manton Center for Orphan Disease Research at Boston Children’s Hospital (BCH), Boston, MA, United States

**Keywords:** prematurity, immunity, T cell, T cell repertoire, immune repertoire, neonate, bronchopulmonary dysplasia, T cell receptor excision circle

## Abstract

Premature infants are known to have immature immune systems compared to term infants; however, the impacts of ex utero immune development are not well characterized. Our previous retrospective clinical review showed prolonged T cell lymphopenia in a subset of extremely premature infants, suggesting that they may have lasting abnormalities in their T cell compartments. We used T cell receptor (TCR) repertoire sequencing to analyze the composition of the T cell compartment in premature and term infants in our NICU. We collected twenty-eight samples from individual subjects and analyzed the number of clonotypes, repertoire diversity, CDR3 length, and V gene usage between groups based on gestational age at birth and postmenstrual age at the time of sample collection. Further, we examined the TCR repertoire in infants with severe bronchopulmonary dysplasia (BPD) and those with abnormal T cell receptor excision circle (TREC) assays. Former extremely premature infants who were corrected to term postmenstrual age had TCR repertoire diversity that was more similar to term born infants than extremely premature infants, supporting normal maturation of the repertoire. Infants with severe BPD did not appear to have increased abnormalities in repertoire diversity. Decreased TCR repertoire diversity was associated with repeatedly abnormal TREC screening, although the diversity was within the normal range for subjects without low TRECs. This study suggests that extremely premature infants demonstrate normal maturation of the T cell repertoire *ex utero*. Further work is needed to better characterize postnatal T cell development and function in this population.

## Introduction

Neonatal sepsis is a leading cause of mortality in preterm newborns (1). Late onset sepsis remains a significant problem throughout the duration of the NICU stay, even as premature infants approach term postmenstrual age. In a retrospective review, we have seen that a subset of extremely premature infants has persistent T cell lymphopenia (TCL) at term postmenstrual age (PMA) (2), which may contribute to this increased sepsis risk. It is known that premature infants are born with decreased T cell counts compared to term-born infants, however the impacts on prematurity on the developing T cell compartment are not well characterized. A flow cytometry-based study examining immune cell populations in premature infants demonstrated that the T cell compartment was the slowest immune cell population to mature as these infants aged (3). Being born prematurely exposes the neonate to a variety of stimuli that would not be encountered if development had continued *in utero*, including higher oxygen exposure, microbial colonization, and numerous metabolic stresses such as having to independently thermoregulate and to digest and absorb nutrition (4). These stressors are known to contribute to the development of disorders of abnormal maturation in premature babies including bronchopulmonary dysplasia (BPD) of the lungs, retinopathy of prematurity, and neurodevelopmental impairment. The impacts of premature birth on the immune system, however, are just beginning to be understood. Indeed, a longitudinal study of extremely premature infants showed that former premature infants who failed to shift their CD4^+^ T cell populations from CD31^-^TNFα^+^ to CD31^+^IL8^+^ T cells around term postmenstrual age experienced a 3.5-fold increase in respiratory complications after NICU discharge (5).

Monitoring immune development in premature infants can be challenging, owing to large blood volumes that are required for certain immune analyses. Advances in flow cytometry and genetics have allowed more precise measurement using smaller aliquots of blood. However, enrolling premature infants in research studies remains fraught with difficulty owing to parental concern about impacts on infants (6, 7). For our initial investigation into T cell maturation in premature infants, we sought to utilize an approach that would allow for minimal risk to infants while still allowing us to gain valuable insights into the T cell compartment. T cell receptor (TCR) repertoire sequencing involves deep sequencing all the T cell receptor sequencing in a population and provides valuable information about the diversity and composition of the T cell compartment. Skewing of the TCR repertoire, leading to decreased diversity and restricted gene usage, is a feature of genetic T cell immunodeficiencies such as severe combined immunodeficiency (SCID) and Wiskott Aldrich Syndrome (WAS) (8–11). TCR repertoire sequencing also can be done using very small volume blood samples, which we hypothesized would lead to high study enrollment.

Other groups have demonstrated that premature infants have a distinct TCR repertoire compared to term neonates. Cord blood analysis from premature infants showed that premature infants have a shorter complementarity determining region 3 (CDR3) length compared with term infants, however differences in the TCR repertoire were less than those seen in the B cell repertoire (12). The fetal TCR repertoire has likewise been shown to be more diverse than that of term babies and adults, and to have a shorter CDR3 (13). Indeed, the diversity of the repertoire continues to diminish throughout the lifespan and diversity correlated with the percentage of naïve CD4^+^ T cells (14). Another study of cord blood repertoires in premature infants demonstrated that extremely premature infants had shorter CDR3 lengths, and that CD8^+^ T cells utilized more public clonotypes (clonotypes that are shared between subjects from the same group) (15). To our knowledge no one has longitudinally analyzed how the TCR repertoire of extremely premature infants develops over time and whether they will converge with their term born counterparts around 40 weeks postmenstrual age.

In addition to being useful for characterizing T cell development in former premature infants, TCR repertoire may also be useful in distinguishing infants who have T cell lymphopenia due to being premature from those who have T cell lymphopenia due to a more critical reason such as an inborn error of immunity (IEI). Newborn screening for SCID using the T cell receptor excision circle (TREC) assay is part of national newborn screening efforts. TREC screening saves lives (16), however, TREC screening is not specific in premature infants (17) and some features of IEI are very challenging to clinically discern from complications of prematurity (18, 19). National reports of TREC screening programs through USIDNET have not detected SCID in any premature infants despite testing being in place for over 10 years (16). Given the incidence of SCID and the number of premature births, this is not statistically likely and supports our hypothesis that diagnoses are being missed. New approaches are needed to enhance diagnostic sensitivity and specificity in premature infants. TCR repertoire may be able to distinguish between T cell IEIs and control patients and could be a useful adjunct to TREC screening in extremely premature infants.

In this study, we analyze TCR repertoire sequencing from premature infants and control term infants collected in a tertiary care NICU. We aimed to determine whether TCR repertoire revealed abnormalities in the T cell compartments of premature born infants (whether at birth or as they matured). Our secondary aim was to examine whether TCR repertoire might be useful in highlighting premature neonates with genetic T cell disorders since T cell lymphopenia is common in this population.

## Methods

### Study Design

The study was approved by the Boston Children’s Hospital IRB (IRB-P00023454) and registered with ClinicalTrials.gov (NCT03433846 “Predictors of Sepsis in Ex-Preterm Infants”). Enrollment criteria was any infant admitted into the NICU, with those less than 37 weeks’ gestation at birth in the premature group and those 37 weeks and older in the term group. No exclusions were made for concurrent diagnoses, as any patients enrolled with potential T cell lymphopenia due to genetic disorders (DiGeorge, Trisomy 18, etc) would be considered positive controls for T cell lymphopenia. Using a standard deviation for TCR repertoire diversity calculated from our previous study (around 0.2), and a desired power of 0.8, we calculated that 7-10 subjects would need to be recruited per group (term and premature) to detect differences in repertoire diversity. Initially we enrolled subjects into groups based on gestational age only, but on enrollment review we realized the postmenstrual ages at sample collection for the premature born cohort varied widely, averaging out to term postmenstrual age with a range of 27 weeks to 68 weeks postmenstrual age. Therefore, we opted to subgroup the premature born infants into 4 cohorts that better accounted for differences in both age at birth and age at sampling: the premature born, premature sampled group (PT) was <30 weeks at birth and less than a month old at sampling; the late preterm (LPT) group was 33-37 weeks at birth and less than a month old at sampling, the premature born, term sampled group (PT-T) was <30 weeks at birth and >37 weeks at sampling, and the late preterm-term (LPT-T) group was born between 33 and 37 weeks and had samples collected after one month of life and beyond 40 weeks postmenstrual age.

For the sub-analysis of subjects with or without severe bronchopulmonary dysplasia (sBPD), we identified a subgroup of infants who developed severe bronchopulmonary dysplasia, characterized by need for continued mechanical ventilation at term postmenstrual age (invasive or non-invasive) (20). All of these infants were within the PT-T group, and the remaining members of the PT-T group, none of whom met the definition for severe BPD, were used as controls for the sub-analysis.

### Sample Collection

Potential study patients were identified by reviewing the medical record. The clinical research team approached patient guardians to inform them of the study and provide information if interested. Those guardians who wished for their baby to participate in the study gave informed consent.

Once a patient was enrolled in the study, a blood sample was obtained. This sample was an additional ~0.5mL of blood drawn for the study at the same time as a blood draw that was obtained for clinical purposes. In this way no blood draws were done solely for the purpose of this study. Samples were obtained in EDTA collection tubes. After the sample was obtained, it was transferred to the research storage facility that day or the following morning (for samples drawn overnight) and stored at -80°C.

### Sample Processing

Frozen samples were processed within three months of collection. Frozen samples were thawed at room temperature, and then RNA was isolated using a Machery-Nagel (Allentown, PA) NucleoSpin RNA Blood, Mini kit according to the manufacturer’s directions. 200μL of the collected sample was used for each analysis, in order to maintain consistent starting volume for each sample. RNA quality control was determined with a Nanodrop to ensure adequate concentration and lack of DNA and protein contamination. RNA was frozen at -80 °C and shipped on dry ice to iRepertoire (Hunstville, AL). iRepertoire performed library preparation and sequencing. Library prep utilized long-read PCR amplification of the human TCR beta chain. Sequencing was done using Illumina MiSeq with v2 500-cycle kit chemistry.

### Data Analysis

iRepertoire provided basic analysis included sequences reads per sample and the raw sequencing data. TCR beta chain reads were mapped using MiCXR version 3.1.13 (21, 22). T cell repertoire analyses of mapped reads were completed using the immunarch package in R version 4.0.3 (23, 24). Clonotypes were normalized by the number of reads per sample per the package default. Graphs were generated using the ggplot2 package (25).

### Data Repository

Raw and processed data was uploaded to Gene Expression Omnibus (GEO), series accession number GSE193637 (26, 27).

### Statistics

Correlation of TCR beta chain reads between duplicate samples from patient P15 for reproducibility assessment was determined using a Pearson correlation. Clonality statistics, gene usage, and diversity estimations were performed using immunarch standard statistical package: for comparisons with 2 groups, the Wilcoxon rank sum test was performed and for comparisons with more than 2 groups, the Kruskal-Wallis test was performed. Adjusted p-values were calculated using the Holm method. Analysis of individual TRBV gene usage differences was done using 2-way ANOVA with multiple comparisons.

## Results

### Subjects and Enrollment

**Figure 1:**
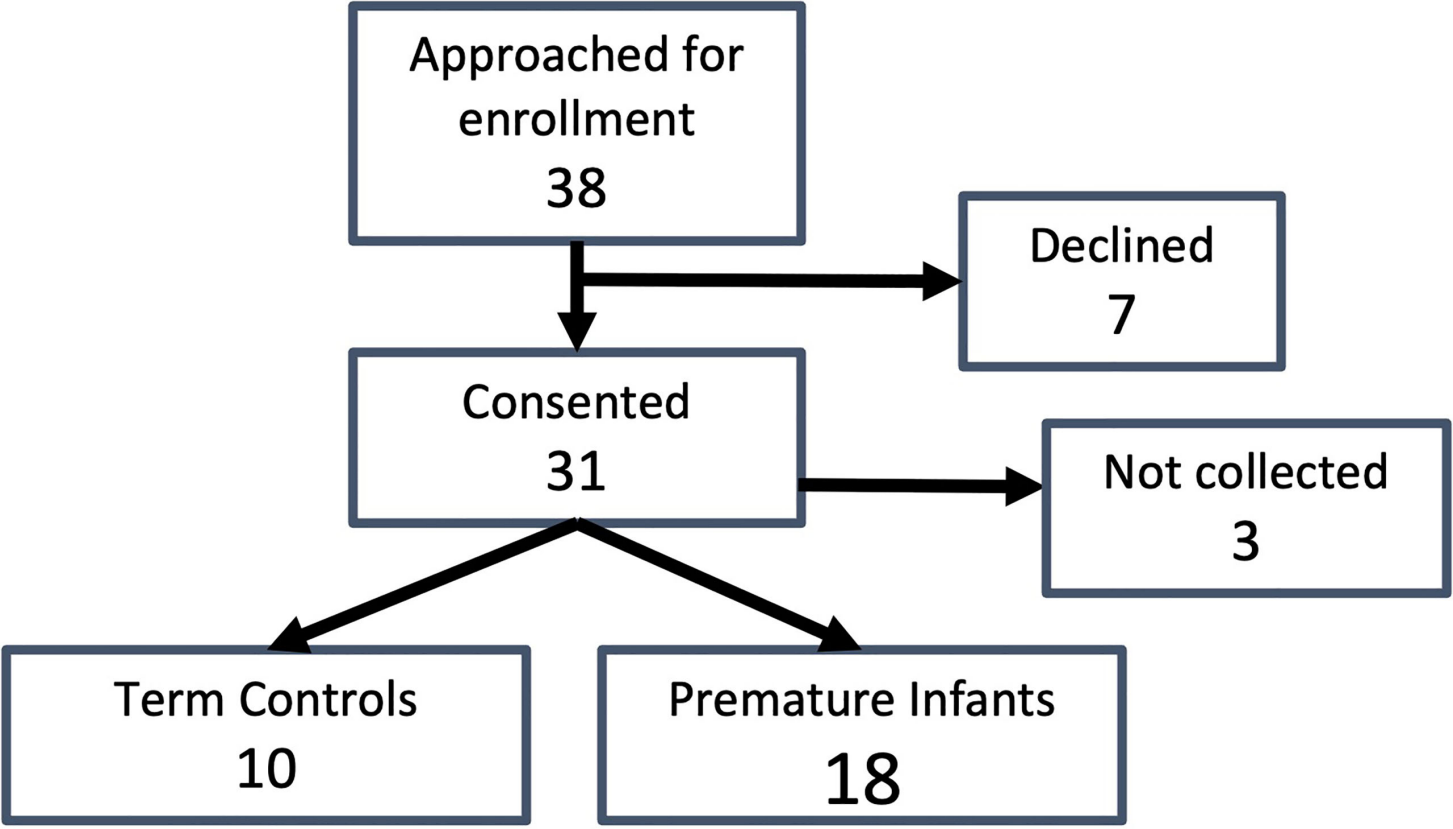
Study enrollment. Total subjects approached for enrollment, number who consented, enrollment numbers by group (premature and term born), and samples collected for each group are indicated.

Thirty-eight guardians were approached for study enrollment and thirty-one consented to study enrollment (81.5%) (**Figure 1**). Three patients were discharged prior to sample collection. Of the twenty-eight patients who had samples collected, eighteen subjects were enrolled in the premature group, while ten subjects were enrolled in the term group.

There was even enrollment by gender for both term born (**Table 1**) and premature born (**Table 2**) subjects. In addition to prematurity, patients in the study had a wide array of diagnoses leading them to be admitted to the NICU. Three of the premature patients had a co-diagnosis of severe bronchopulmonary dysplasia with pulmonary hypertension. None of the enrolled patients was diagnosed with an immunologic disorder, although four of them had low TRECs during their admission. Four patients had long gap esophageal atresia, the result of our surgical team having world-renowned expertise in managing this condition. Infections were minimal in the cohort: T20 had a scalp infection and urosepsis due to *E. Cloacae* before the study period, P20 had sepsis with *E. cloacae* two weeks prior to sample collection, and P21 had a *Pseudamonas* chest tube infection a couple of weeks prior to the study sample. The average gestational age of the infants in the term group was 39 weeks (range 37 1/7 to 40 4/7) and the average corrected age at the time of sampling was 42 3/7 weeks. The average age of the all premature infants was 30 3/7 (range 24 2/7 to 36 3/7) and the average age at sample collection was 43 6/7 weeks (range 27 4/7 to 68 5/7).

**Table 1:** Subject characteristics, term born subjects.

Subject ID	Sex	GA	Additional Diagnoses	PMA at Sample
T09	M	37.1	Hyperbilirubinemia	38.4
T11	F	40.0	Hyperammonemia	41.7
T12	F	39.1	Tetrology of Fallot and Imperforate Anus	40.1
T13	M	38.1	Myelomeningocele, Chiari II, Hydrocephalus	41.1
T14	M	40.0	Choking spells	42.3
T16	M	38.3	VSD, Volvulus	42.1
T17	M	39.3	CF, Meconium Ileus	41.0
T18	F	38.9	Pulmonary HTN, meconium aspiration	39.7
T19	M	39.0	Vein of Galen Malformation, heart failure	44.1
T20	F	40.6	Tetrology of Fallot, cutis aplasia	45.4
**AVERAGE**		**39**		**41.6**

PMA, postmenstrual age.

**Table 2:** Subject characteristics, premature born subjects.

PREMATURE BORN					
Subject ID	Sex	GA	Additional Diagnoses	PMA at Sample	Additional Cohort
**PT**					
P17	M	26.1	NEC, mild BPD	29.7	TRECs
P23	M	27.0	Intestinal perforation	30.7	TRECs
P25-1	M	26.1	Intestinal perforation, BPD	27.6	TRECs
P27	F	29.0	Tracheal Ring	32.6	TRECs
**AVERAGE**		**27.1**		**30.1**	
**LPT**					
P10	M	36.9	Respiratory/renal failure/hydronephrosis	37.1	
P14	F	33.4	Vein of Galen, Pulm HTN	35.3	
P26	M	34.9	Gastroschisis	38.1	
P28	M	36.7	Posterior Urethral Valves	38.0	
**AVERAGE**		**35.5**		**37.1**	
**PT-T**					
P12	F	29.6	NEC, mild BPD, IUGR	47.6	BPD control
P13-1	F	25.3	Severe BPD, pHTN	54.0	Severe BPD
P18	M	27.4	Esophageal Stenosis	65.7	BPD control
P20-2	F	24.3	Severe BPD, pHTN	41.0	Severe BPD
P22	M	26.0	Severe BPD, pHTN	68.7	Severe BPD
P29	F	26.7	Long Gap EA	52.3	BPD control
**AVERAGE**		**26.6**		**54.9**	
**LPT-T**					
P15-A	F	36.9	Thrombocytopenia, Sinus Vein Thrombosis	41.0	
P16-1	F	32.9	Long gap EA/TEF	54.0	
P21-1	F	33.6	Long gap EA, mediastinitis	51.4	
P24-1	M	36.6	Long Gap EA	42.9	
**AVERAGE**		**35.0**		**47.3**	

Sub analysis groups are color coded with red samples included in the premature sub analysis, blue samples in the BPD sub analysis, and green in the TREC sub analysis. NEC, necrotizing enterocolitis; BPD, bronchopulmonary dysplasia; TE, tracheoesophageal; PT, extremely or very premature baby with sample within the first month of life; LPT, late premature born infant with sample collected during the first month of life; PT-T, premature baby with sample at term; LPT-T, late premature baby with sample at term; PMA, postmenstrual age; TREC, T cell receptor excision circle.

When broken down into subgroups, the premature sample group (PT) had an average gestational age (GA) of 27 weeks and an average PMA at sample collection of 30 weeks. The late premature (LPT) group had an average GA of 35 weeks and an average PMA at collection of 37 weeks. The premature born, now term PMA group (PT-T) has an average GA of 26 weeks and an average PMA at collection of 54 weeks, and the late premature born, now term PMA group (LPT-T) had an average GA of 35 weeks and a PMA at sampling of 47 weeks.

### Sequencing Depth and Quality Control

The total reads per sample is shown in **Table S1**. Despite a small volume of blood for the initial samples (0.2mL), TCR repertoire sequencing was robust with read counts ranging from 802,622 to 1,637,609 (average 1,160,478). To test reproducibility within our study we also collected duplicate samples from one patient on the same day (T15-A and T15-B) and sequenced both samples to examine how much overlap there was in the repeated independent samples (**Figure S1**). Overall, the two independent samples were highly concordant.

### Premature Infants Had More Clonotypes and Increased Diversity Compared to the Term Born Cohort

**Figure 2:**
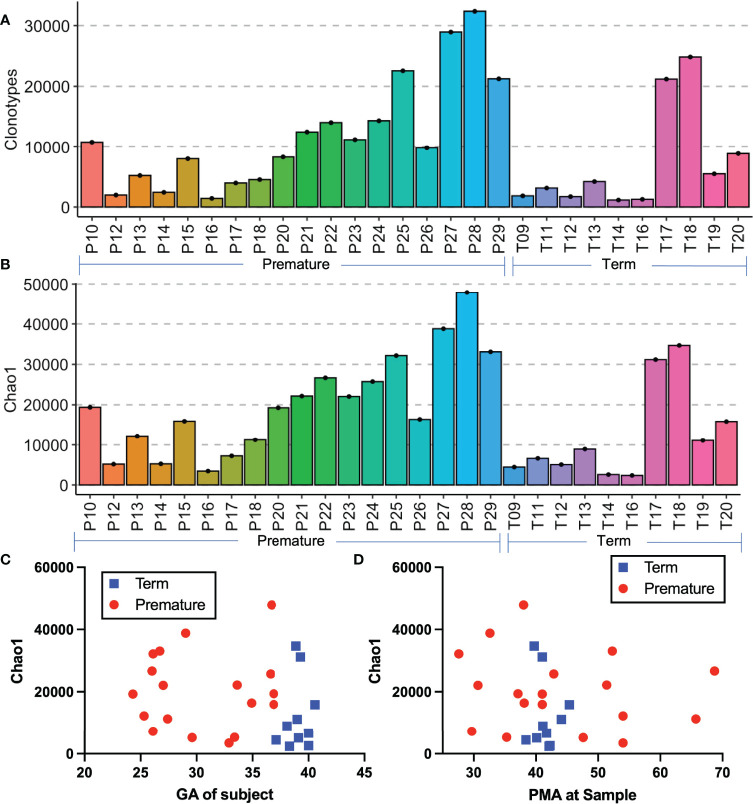
Comparison of premature and term born subjects. Histograms of the number of clonotypes **(A)** and Chao1 richness **(B)** of all samples. Depicted in **(C, D)** are the scatter plots of the Chao1 richness by gestational age at birth **(C)** and by postmenstrual age at sample **(D)**.

Clonotypes are a combined sequence of the V gene and J genes utilized by a TCR in combination with the nucleotide sequence of the hypervariable region of complementarity determining region 3 (CDR3), which contains nucleotide insertions and mutations which may vary from the germline sequence as the TCR rearrangements create additional sequence diversity in order to generate antigen specificity (28, 29). Premature born infants had more clonotypes on average than term born infants (**Figure 2A**), however the distribution of clonotypes was similar between the premature born and term born subjects. Chao1 richness, an estimation of species richness based on abundance, was also evenly distributed between the premature born and term born subjects (**Figure 2B**). We also examined the relationships between gestational age at birth (**Figure 2C**) and postmenstrual age at sampling (**Figure 2D**) and the Chao1 richness, and found that there was no linear association between either variable and the sample diversity.

### Former Extremely Premature Born Infants Have Similar Diversity and Clonal Expansion Compared to Term Infants

**Figure 3:**
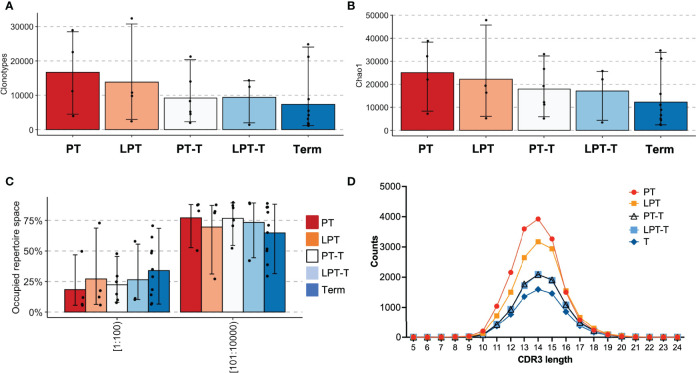
Analysis of premature born infants by age at testing. Extremely premature infants who were within a month of birth at the time of testing (PT) were compared with late premature infants who were within a month of birth at the time of testing (LPT), former extremely premature infants who were term postmenstrual age at testing (PT-T), former late premature infants who with term PMA at time of testing (LPT-T) and term infants who were term or post-term at testing (term). Clonotype counts are shown in **(A)**, Chao1 diversity is shown in **(B)**, clonal expansions of the top 100 clonotypes by percentage of the repertoire and the next 100-10,000 most abundant clonotypes are in **(C)**. The distribution of lengths of CDR3s for each group was averaged and is shown in **(D)**.

One of the goals of this project was to examine the impacts of premature birth on T cell development. We therefore subdivided the premature subjects into those who were still premature at the time of the sample collection (preterm/preterm, PT - P17, P23, P25, P27), those who were late preterm at the time of sample collection (LPT - P10, P14, P26, P28), those who were born premature but were term or post term PMA at the time of the sample (preterm/term, PT-T - P18, P20, P22, P29), and those who were born late preterm and were term or post term PMA at the time of sample (LPT-T, P15, P16, P21, P24). We compared these to term born subjects with samples collected at term or post term (term – all subjects in the term group T9-T20). The mean number of clonotypes was greatest for the PT group, less for the LPT group, and lower for PT-T, LP-T, and term groups (**Figure 3A**). The means were similar for all of the term PMA collected groups, regardless of GA (PT-T, LPT-T, and term). The diversity by Chao1 richness followed a similar trend (**Figure 3B**). The mean proportion of the repertoire occupied by the top 100 most abundant clonotypes was highest in the term group and lowest in the PT group (**Figure 3C**), meanwhile CDR3 length distribution was similar between all subgroups (**Figure 3D**).

**Figure 4:**
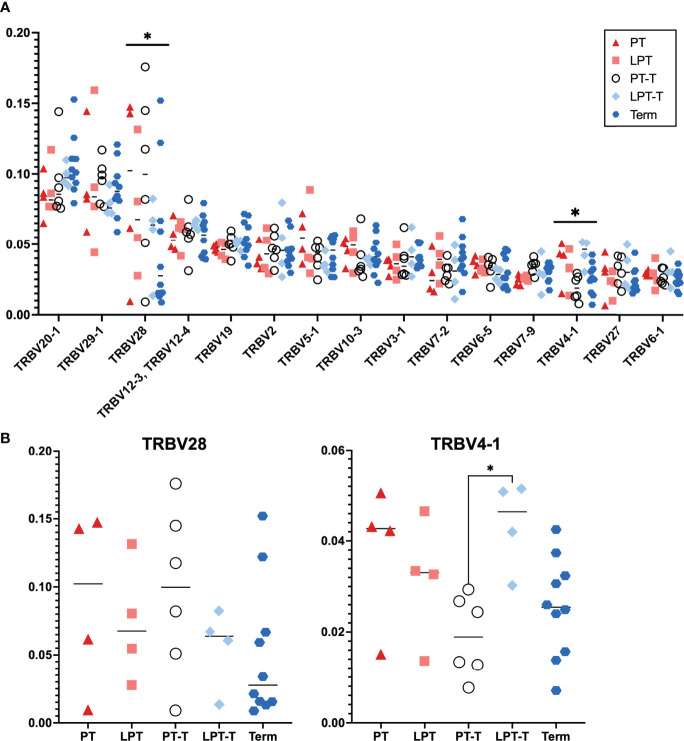
T cell receptor beta chain V gene (TRBV) usage for PT, LPT, PT-T, LPT-T and TT groups. **(A)** Distribution of most abundant TRBV for all samples. Displayed are the 15 most abundant TRBV genes used in the population. Analysis *via* 2-way ANOVA with Tukey’s multiple comparison. **(B)** Analysis of individual TRBV genes that were different between subgroups in **(A)** For **(B)**, statistical analysis was by 1 way ANOVA with Tukey’s multiple comparison. An asterisk indicates p < 0.05.

Analysis of TCR Vβ (TRBV) gene usage between these subsets showed that overall V gene usage was similar between groups (**Figure 4A**, shows those V beta chains with average abundance >1%)). There was a significant difference in expression of TRBV28 and TRBV4-1 by 2-way ANOVA with Tukey’s test for multiple comparisons when all groups were assessed. When we considered expression of these V beta genes independently (**Figure 4B**) and analyzed for significance using one way ANOVA with Tukey’s multiple comparison, only TRBV4-1 expression was significantly different between samples, with the LPT-T group significantly different than the PT-T group. Overall, TRBV gene usage was very similar between groups.

**Figure 5:**
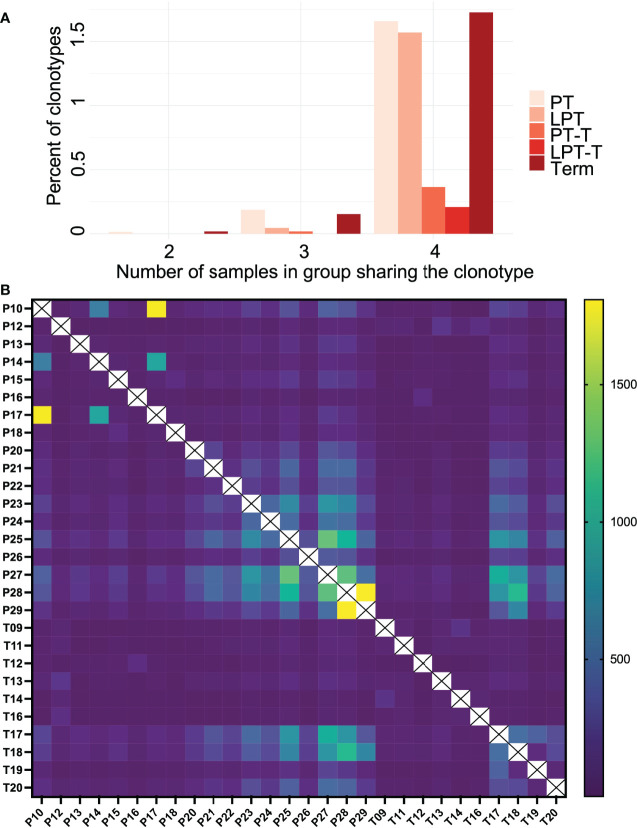
Public clonotype usage for PT, LPT, PT-T, LPT-T and TT subgroups. **(A)** Depicted are the percentage of clonotypes shared within group members (y-axis), and the number of group members sharing the overlapping clonotypes (x-axis). **(B)** A heat map indicating clonotype overlap between samples.

We also examined public clonotype usage in premature subgroups versus term infants (**Figure 5A**). About 0.5-2% of clonotypes were publicly shared by the subjects in each group, and the PT, LPT, and term groups had a higher number of subjects that shared the same clonotypes (mostly 4 or more) compared to the PT-T and LPT-T groups, although this was not statistically significant. Clonotype overlap between individual samples is represented in a heatmap in **Figure 5B**.

### Subjects With Severe BPD Did Not Demonstrate Abnormal Repertoire Development

**Figure 6:**
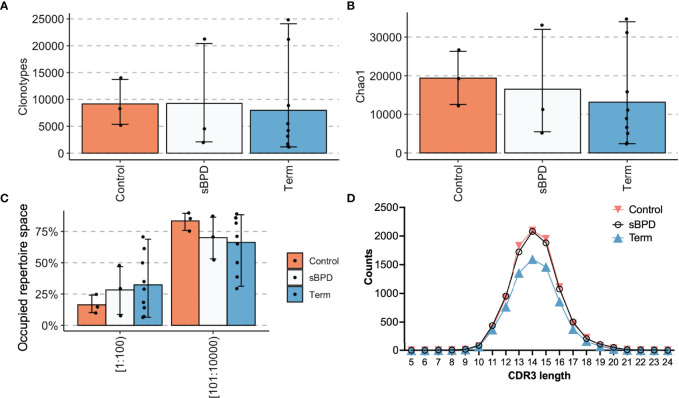
Sub analysis of premature infants who developed severe BPD compared with those who did not. Extremely premature infants who developed severe BPD (sBPD) were compared with former extremely premature infants who did not develop severe BPD (Control) and term infants (Term). Clonotype counts are shown in **(A)**, Chao1 diversity is shown in **(B)**, clonal expansions of the top 100 clonotypes by percentage of the repertoire and the next 100-10,000 most abundant clonotypes are in **(C)**. The distribution of lengths of CDR3s for each group was averaged and is shown in **(D)**.

Mouse models of BPD have demonstrated thymic atrophy (30), which may affect the T cell compartment. To determine whether former premature infants with severe BPD experience abnormalities in TCR repertoire, we specifically analyzed the repertoires of the three former premature patients with severe BPD (P13, P20, P22) against three former premature infants who did not have moderate or severe BPD (P12, P18, P29) and term control patients (T09-T20). Premature born infants without BPD had similar numbers of clones and clonotypes (**Figures 6A**, **B**) to the control groups and had a similar proportion of the repertoire occupied by the top 100 most abundant clonotypes (**Figure 6C**). CDR3 length distributions were similar between the groups (**Figure 6D**). Overall, the infants in the former premature severe BPD group and the former preterm infants without BPD were very similar and were not statistically different than term infants.

### Repeated Abnormal TREC Screening Was Associated With Lower TCR Repertoire Diversity

**Figure 7:**
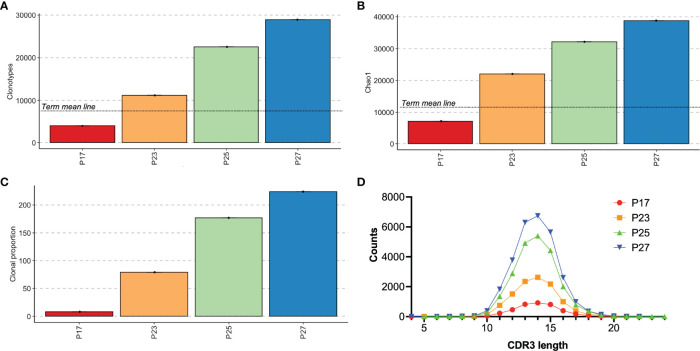
Analysis of premature infants had positive TREC testing. Individual results for subjects who had abnormal TREC screening. Clonotype counts are shown in **(A)**, Chao1 diversity is shown in **(B)**, clonal expansions of the top 100 clonotypes by percentage of the repertoire and the number of clonotypes occupying 10% of the repertoire are shown in **(C)**. **(A, B)** include a mean line of the term control group from **Figure 3** for comparison. The distribution of lengths of CDR3s for each subject is shown in **(D)**.

We next were curious to see whether TCR repertoire might help to differentiate premature infants at higher risk for primary immunodeficiency compared with those who have abnormal TRECs just from being premature. In keeping with the known problem that TREC screening has high rates of false positive in premature babies (16, 31, 32), all of our extremely premature babies who were still premature at the time of sample collection (PT group - P17, P23, P25, P27) also had low TRECs on their initial newborn screens. The newborn screening lab in Massachusetts reports low TRECs as <252 copies/μL, and specific TREC levels are not provided for infants with levels below the threshold. The TCR repertoire of the four infants who had low TRECs on newborn screening demonstrates that one subject had decreased clonotypes and decreased diversity compared to the other subjects (**Figures 7A-C**), while CDR3 lengths were similarly distributed (**Figure 7D**). That subject, P17, was born at 26 1/7 weeks and was 25 days old at the time of the sample collection. The patient had severe necrotizing enterocolitis (NEC) and was the only subject in the study that flagged for low TRECs twice during admission – once at 14 days of life (11 days before the sample) and once at 28 days of life (3 days after sample collection). The other three subjects with low TRECs (P23, P25, P27) all had normal TREC levels on their next newborn screen following study sample collection and had more diverse repertoires with at least 10,000 clonotypes in the sample. P17 had developed surgical NEC at day of life 5 with subsequent hypotension requiring pressors, laparotomy, and placement of a diverting ileostomy. He was intubated and on stress dose steroids at the time of the first newborn screen. He came off steroids before the study sample collection and had a repeat newborn screen three days after the study sample was collected that still showed low TRECs. The next TREC screen at 1.5 months of age was within normal limits. He subsequently recovered, had a bowel re-anastomosis, and was discharged to home eventually on room air. He is followed at our continuity clinic and is growing well and has not had issues with recurrent infections. His most recent absolute lymphocyte count was in the normal range.

We also reviewed the clinical records to determine whether any of the subjects in the study, whether TRECs were abnormal or not, had T cell lymphopenia. Only two subjects in the study had immunologic flow cytometry done, P14 and P13. P14 had flow done about two weeks after the sample was collected, and it showed significant T cell lymphopenia with an absolute CD3 count of 408 cells/mm3 (age-based ref range 2500-5600). The patient was very ill at that time and unfortunately died from complications related to vein of Galen malformation a couple of weeks later. Another subject, P13, also had immune flow cytometry done that showed mild T cell lymphopenia with an absolute CD3 count of 1603 cells/mm3 (ref range 1900-6200). This patient had comparable clonal expansion to the group average. There were not enough subjects with T cell counts to make any conclusions about how T cell lymphopenia compares to TCR repertoire diversity or clonal expansion.

## Discussion

This study builds upon existing literature probing the long-term immunologic impacts of prematurity. We and others have demonstrated that a subgroup of former premature infants has lasting abnormalities in the T cell compartment (2, 5). Studies of cord blood samples have shown that premature infants have less mature TCR repertoires compared to term infants, including shorter CDR3 lengths and greater diversity (12, 15). We therefore sought to determine whether these differences in the premature immune repertoire also persist over time in premature infants as they grow and approach term postmenstrual age. Another recent study also examined cord blood TCR repertoire and compared premature infants with term infants, however the gestational age range for the premature babies was 32-35 weeks, so our population included significantly more extremely premature infants (12). However, CDR3 lengths in our premature population were not appreciably different than term infants. This could be because we sampled peripheral blood rather than cord blood, and that the samples were not immediately postnatal. It may be that the peripheral blood repertoire in the infants is not well represented by cord blood, or, more likely, that the repertoire begins to change quickly after birth. Mathematical modelling of the repertoire over the life course suggests that the majority of TCR expansions occur during the perinatal time period, with subsequent changes occurring more slowly throughout the rest of the lifespan (33, 34).

The finding that the PT-T and LPT-T groups had similar numbers of clonotypes and repertoire diversity compared with term infants suggests that repertoire maturation in premature born infants seems to progress normally compared to term-born infants at the same PMA. While our sample size is small, our hypothesis that extremely premature infants may have delayed or altered T cell development postnatally was not supported by the data. It has been previously reported that diversity correlates with naïve CD4^+^ T cell populations (14), while public clonotype usage had an association with CD8+ T cell populations (15), so it is possible that by not separating these cell populations we may have lacked the resolution to detect population-specific shifts. Former premature infants do demonstrate increased clinical susceptibility to viral infections after discharge from the hospital, including respiratory syncytial virus, rhinovirus, and adenovirus (35). Abnormal T cell maturation could contribute to this susceptibility. Studies in humans have shown that abnormal maturation of the CD4^+^ T cell population can be linked with respiratory complications in former premature infants (5). Others have reported that the relative proportions of naïve, memory, and effector T cells are similar between extremely premature infants and term infants (36), however the percentages of CD8 and T regulatory cells are different between these two populations. Given the small blood sample sizes used for this study, we were not able to assess sub-populations of T cells. In future studies it will be important to separate CD4^+^ and CD8^+^ T cell populations for sub analysis, something that was not possible in this study.

Some small differences in TRBV gene expression were seen in the repertoires, however overall TRBV gene expression was quite similar between the premature subgroups and term infants. Other reports have shown differential usage of TCRβ V4 family genes in premature versus term infants in CD8 T cells (15), and we did detect differences in expression of TRBV4-1 in our cohort, however this was quite subtle and only different between two of the 5 subgroups.

Assessment of shared or public clonotypes between the groups showed that the PT, LPT, and term groups tended to have more shared clonotypes while the PT-T groups and LPT-T groups had fewer. This might reflect the PT-T and LPT-T groups having more postnatal time to encounter antigens and alter their repertoires to respond to these stimuli compared to the PT, LPT, and term groups, who had samples collected closer to birth.

One of the limitations of the study is that we had conceived of using all premature infants <37 weeks in the initial enrollment groups, but as we examined the enrollment demographics we realized we had a wide range in postmenstrual age of the premature group and it was more physiologically relevant to subgroup these infants based on both age at birth (very and extremely premature compared to late premature) and postmenstrual age when the sample was drawn (within one month of life or after term postmenstrual age). We did this prior to analyzing the data and was not due to something we noted on data analysis, but a decision we made before we started analyzing the data. Ideally, we would have done patient recruitment with the same definitions and that would have boosted our n number for the premature subgroups.

Another limitation was the very small blood volumes we utilized. While we had robust samples with high numbers of clones detected and also showed good reproducibility with the paired samples from P15, it is possible that such a small sample failed to reveal more subtle differences between the study populations.

Because we are an all-outborn, surgical and subspeciality care NICU, premature infants are typically only transferred to our unit if they have a surgical issue or a significant complication or co-diagnosis. For that reason, we were not able to get samples at the time of birth for most of the premature infants. This information may have been informative when trying to compare our results to previous premature TCR repertoire studies which have all used cord blood samples.

The number of patients enrolled in our study (28 total subjects) is a strength, as a majority of TCR repertoire studies utilize *n* numbers of 10 or fewer. Another unique aspect of our study is the use of blood samples from postnatal patients rather than cord blood samples, which are the most frequently utilized samples in neonatal immune studies owing to the large amount of blood available and ease of obtaining it. While cord blood samples may be convenient, it is hard to know how much they reflect the *ex utero* neonate, and studies have shown rapid chnges in peripheral blood populations after birth (37).

The lack of any appreciable difference between former premature infants with BPD and without BPD does not support the concept that BPD selectively impairs thymic development. Our study size was small, thus, was not powered to address this as a primary outcome. Additional clinical studies examining the peripheral T cell compartment for infants with severe BPD will be helpful in understanding this paradigm.

An additional sub-hypothesis of this study was that analysis of the TCR repertoire might be useful in distinguishing premature infants with low TRECs due to prematurity-related lymphopenia versus premature neonates with SCID and other T cell immunodeficiency disorders. Analysis of infants with low TRECs showed good clonotypic diversity for three patients, and these patients all had normal TRECs with the next newborn screen. One patient who was very severely ill at the time of the study collection had decreased repertoire diversity with clonal expansion and the next TREC assay was also abnormal, but TRECs eventually normalized two weeks after the sample collection and the patient recovered and is now growing and without recurrent infections. Our data are not conclusive as to whether TCR repertoire assessment would be a useful adjunct in screening for SCID in premature infants.

In sum, our study examined TCRβ receptor repertoire for total T cell populations in peripheral blood samples from premature born and term born patients in a tertiary care NICU. We found that premature infants had a trend toward increased TCR repertoire diversity as previously reported, and that as extremely premature infants aged their repertoires became similar to term born counterparts at the same postmenstrual age. This suggests that repertoire development proceeds normally in extremely and late premature infants despite early exposure to the extrauterine environment.

## Data Availability Statement

The datasets presented in this study can be found in online repositories. The names of the repository/repositories and accession number(s) can be found in the article/**Supplementary Material**.

## Ethics Statement

The studies involving human participants were reviewed and approved by Boston Children’s Hospital Institutional Review Board. Written informed consent to participate in this study was provided by the participants’ legal guardian/next of kin.

## Author Contributions

SM carried out the data analysis and generated the figures for the paper. MS and RK did the patient recruitment and sample collection. VY oversaw the clinical team and helped to write the IRB and generate the ClinicalTrials.gov study registration. AO conceived and designed the study, obtained funding, isolated the sample RNA, oversaw study implementation, and wrote the manuscript. All authors contributed to the article and approved the submitted version.

## Funding

This work was supported by Boston Children’s Hospital Office of Faculty Development Career Development Award (SM), and the Manton Center Innovation Fund (AO).

## Conflict of Interest

The authors declare that the research was conducted in the absence of any commercial or financial relationships that could be construed as a potential conflict of interest.

## Publisher’s Note

All claims expressed in this article are solely those of the authors and do not necessarily represent those of their affiliated organizations, or those of the publisher, the editors and the reviewers. Any product that may be evaluated in this article, or claim that may be made by its manufacturer, is not guaranteed or endorsed by the publisher.
