# Supplementary figures and images for: Premature Infants Have Normal Maturation of the T Cell Receptor Repertoire at Term
Source: Front Immunol. 2022 May 30;13:854414. doi: 10.3389/fimmu.2022.854414 (PMC9189380; doi:10.3389/fimmu.2022.854414)

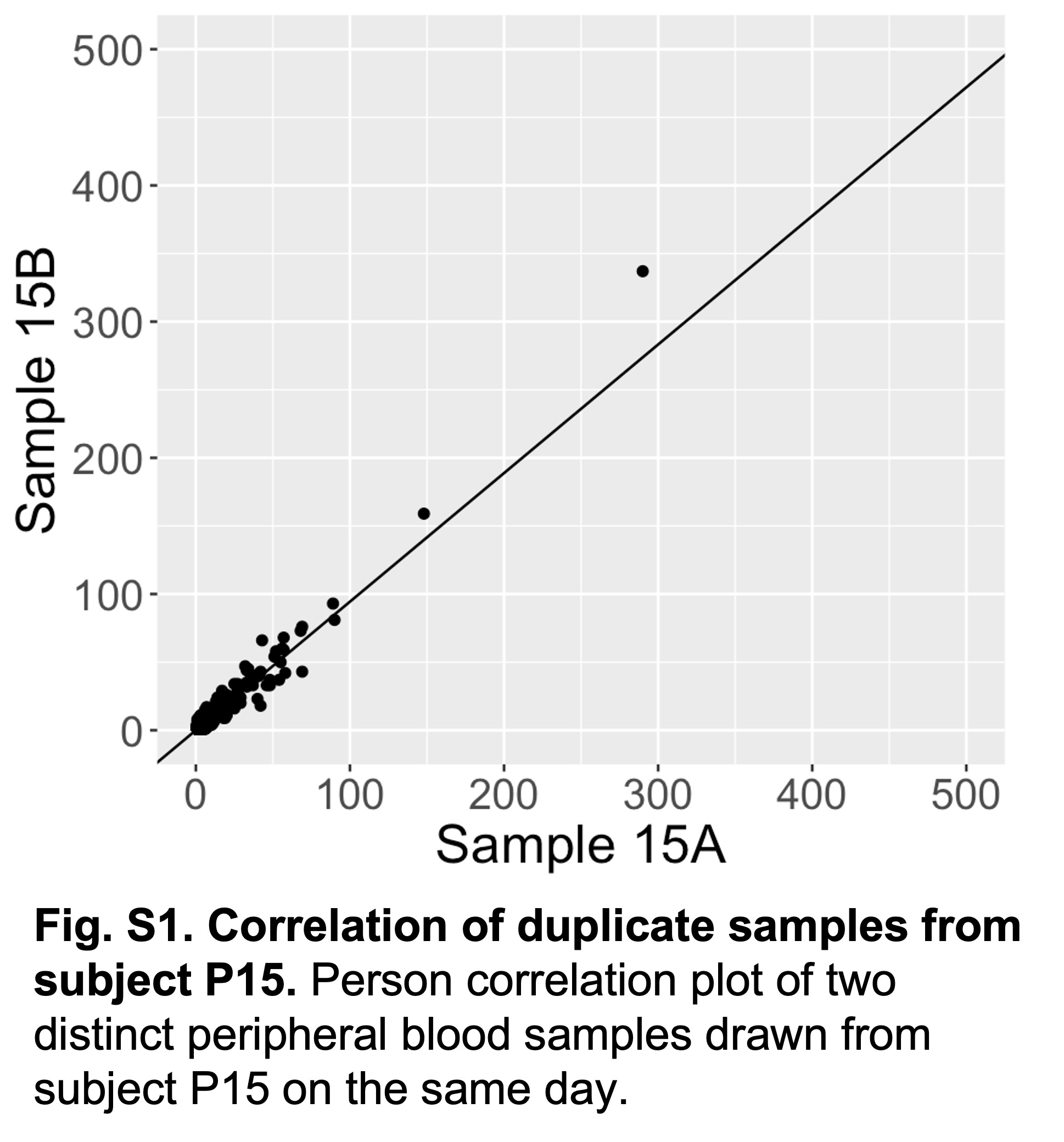

Supplement: Supplementary file 1 [file Image_1.jpeg]
